# Nab-Paclitaxel in combination with Cisplatin Versus Docetaxel Plus Cisplatin as First-Line Therapy in Non-small Cell Lung Cancer

**DOI:** 10.1038/s41598-017-11404-9

**Published:** 2017-09-07

**Authors:** Yi Chen, Jinyu Li, Shixue Chen, Yibao Zhang, Yi Hu, Guoqing Zhang, Xiang Yan, Shunchang Jiao

**Affiliations:** 1 School of Clinical Medicine, Tsinghua University, Beijing, China; 2 Department of Medical Oncology, Chinese PLA General Hospital, Beijing, China; 3 Key Laboratory of Carcinogenesis and Translational Research (Ministry of Education/Beijing), Department of Radiotherapy, Peking University Cancer Hospital & Institute, Beijing, China

## Abstract

Albumin-bound paclitaxel (nab-PC) and docetaxel both produced favorable efficacy and safety as first-line therapy in advanced non-small cell lung cancer (NSCLC). However, the comparison between nab-PC and docetaxel remained unclear until now. This retrospective study aimed to compare the efficacy and safety of nab-PC/cisplatin with docetaxel/cisplatin as first-line therapy in advanced NSCLC. 271 patients with advanced NSCLC, who received either nab-PC (55 patients) or docetaxel (216 patients) were reviewed from 2012 to 2016. The primary endpoint was objective overall response rate (ORR). The secondary endpoints were disease control rate (DCR), progression-free survival (PFS), overall survival (OS) and safety profiles. Nab-PC presented a significantly higher ORR than docetaxel (47.3% *vs* 31.9%; P = 0.033). The difference of ORR was more significantly remarkable in patients with squamous histology (58.3% *vs* 29.0%; P = 0.007). Additionally, the DCR of nab-PC was significantly higher than docetaxel. Patients in nab-PC group had a trend toward improved PFS and OS compared with patients in docetaxel group, but this didn’t reach statistical significance. Grade ≥ 3 neutropenia was less in nab-PC group, while Grade ≥ 3 anemia and thrombocytopenia were less in docetaxel group. Nab-PC/cisplatin as first-line therapy, produced significantly higher efficacy and reduced neutropenia than docetaxel/cisplatin in advanced NSCLC.

## Introduction

Lung cancer is the leading cause of cancer-related death throughout the world, within which NSCLC accounts for approximately 85% of all lung cancer cases^1, 2^. More than half of the patients with NSCLC present with advanced disease at the time of diagnoses, and systemic chemotherapy of platinum-based doublets is generally considered to be the current standard care for those patients in first-line setting, due to its efficient antitumor activity and acceptable toxicity^3^.

Docetaxel plus cisplatin is a commonly used taxane-platinum combination in the management of advanced NSCLC. They have been demonstrated to be effective against previously-untreated advanced NSCLC. Results of a large phase III trial^4^ found that docetaxel plus cisplatin resulted in a more favorable ORR (32% *vs* 25%, respectively; P < 0.05) and survival (11.3 *vs* 10.1 months, respectively; P < 0.05) than vinerelbine plus cisplatin. Another randomized multicenter phase III trial^5^ demonstrated that docetaxel plus cisplatin was significantly superior to vindesine plus cisplatin in terms of ORR (37% *vs* 21%, respectively; P < 0.05) and OS (11.3 *vs* 9.6 months, respectively; P < 0.05). In general, previous studies^4–14^ indicated that docetaxel plus cisplatin regimen as first-line therapy yielded 29% to 52% ORR, 4 to 7 months of PFS and 8 to 17 months of OS in advanced NSCLC.

Nanoparticle albumin bound (nab) paclitaxel (nab-PC), a solvent-free, nanometersized albumin-bound paclitaxel particle, is invented for avoidance of the toxicities related to polyethylated castor oil. It is administered as a colloidal suspension of 130-nm format, taking advantage of the unique properties of albumin, therefore, allowing higher doses of infusion of paclitaxel than the doses of standard paclitaxel therapy and no premedication. Nab-PC, as a novel agent produced superior antitumor activity and safety in NSCLC^15–24^. Additionally, it plays an important role in many other kinds of malignancies, including gastric carcinoma, melanoma, pancreatic cancer and especially breast cancer^25–35, 39^. In a preclinical study, Desai *et al*.^36^ reported that the efficacy of nab-PC was significantly improved compared with docetaxel in multiple tumors xenograft. Moreover, in advanced breast cancer, a randomized, multicenter study^37^ reported that nab-PC/carboplatin regimen significantly improved the PFS by 5.4 months compared with docetaxel/carboplatin. However, up to now, the comparison of efficacy and safety between nab-PC and docetaxel in advanced NSCLC still remains unclear, thus, it is imperative to address this. The present study aimed to directly compare the efficacy and safety of weekly nab-PC/cisplatin q3w with docetaxel/cisplatin q3w in first-line setting in patients with advanced NSCLC, trying to provide some results as a basis for future prospective trials.

## Results

### Patients

A total of 271 patients −55 patients in nab-PC/cisplatin group and 216 patients in docetaxel/cisplatin group- were included from 2012 to 2016. The median age was 58 years (IQR: 51–65) and 90% of patients were younger than age 70 years. Most of the patients were male, smokers and had stage IV disease. The general characteristics of the patients were shown in Table 1. The baseline characteristics of the two groups were well balanced.

**Table 1:** Demographics of patients.

Clinical characteristics	Nab-PC (N = 55)	Decetaxel(N = 216)	All (N = 271)
	No.(%)	No.(%)	No.(%)
**Age(years)**			
Median	59	57	58
IQR	52–66	50–65	51–65
<70	50(90%)	197(91.2%)	247(91.1%)
≥70	5(10.0%)	19(8.8%)	24(8.9%)
**Sex**			
Male	45(81.8%)	180(83.3%)	225(83.0%)
Female	10(18.2%)	36(16.7%)	46(17.0%)
**Smoking**			
Yes	44(80%)	151(69.9%)	195(72.0%)
No	11(20%)	65(30.1%)	76(28.0%)
**ECOG PS**			
0	18(32.7%)	65(30.1%)	83(30.6%)
1	37(67.3%)	151(69.9%)	188(69.4%)
**Histology**			
Squamous cell carcinoma	24(43.6%)	93(43.1%)	117(43.2%)
Adenocarcinoma	28(50.9%)	112(51.9%)	140(51.7%)
Large-cell carcinoma	2(3.6%)	4(1.9%)	6(2.2%)
Other	1(1.8%)	7(3.2%)	8(3.0%)
**Clinical Stage**			
IIIB	20(36.4%)	73(33.8%)	93(34.3%)
IV	35(63.6%)	143(66.2%)	178(65.7%)
**Prior Therapy**			
Surgery	9(16.4%)	26(12.0%)	35(12.9%)
Radiation therapy	1(1.8%)	3(1.4%)	4(1.5%)
Ajuvant Chemotherapy	4(7.3%)	10(4.6%)	14(5.2%)

Abbreviations: ECOG PS, Eastern Cooperative Oncology Group Performance Status; Nab-PC, nanoparticle albumin bound paclitaxel; IQR, inter-quartile range.

### ORR

Two patients (3.6%) achieved CR and 24 (43.6%) achieved PR in nab-PC group and 1 patient (0.5%) had CR and 68 (31.5%) had PR in docetaxel group (Table 2). The nab-PC indicated a significantly higher ORR than docetaxel (47.3% *vs* 31.9%; odds ratio, 1.910; 95% CI, 1.046 to 3.486; P = 0.033). Furthermore, subgroup analysis suggested a significant improvement of ORR for nab-PC versus docetaxel in patients with squamous histology (58.3% *vs* 29.0%; odds ratio, 3.422; 95% CI, 1.355 to 8.646; P = 0.007), while the ORR was comparable between nab-PC and docetaxel in patients with non-squamous histology (38.7% *vs* 34.1%; odds ratio, 1.218; 95% CI, 0.540 to 2.747; P = 0.634).

**Table 2:** Response rates for the patients with advanced NSCLC.

Response rates	Nab-PC(N = 55)	Decetaxel(N = 216)
	No.(%)	No.(%)
**Total population**		
Overall response	26(47.3%)	69(31.9%)
Complete response	2(3.6%)	1(0.5%)
Partial response	24(43.6%)	68(31.5%)
Stable disease	23(41.8%)	70(32.4%)
Progressive disease	6(10.9%)	77(35.6%)
**Squamous Subset**	n = 24	n = 93
Overall response	14(58.3%)	27(29.0%)
**Nonsquamous Subset**	n = 31	n = 123
Overall response	12(38.7%)	42(34.1%)

Abbreviations: Nab-PC, nanoparticle albumin bound paclitaxel.

### DCR

In the present study, 26 patients(47.3%) had response and 23 patients (41.8%) had stable disease in nab-PC group. In docetaxel group, 69 patients (31.9%) had response and 70 patients (32.4%) had stable disease. The nab-PC indicated a significantly higher DCR than docetaxel (89.1% *vs* 64.4%; odds ratio, 4.524; 95% CI, 1.854 to 11.042; P = 0.000).

### Progression-Free Survival

**Figure 1:**
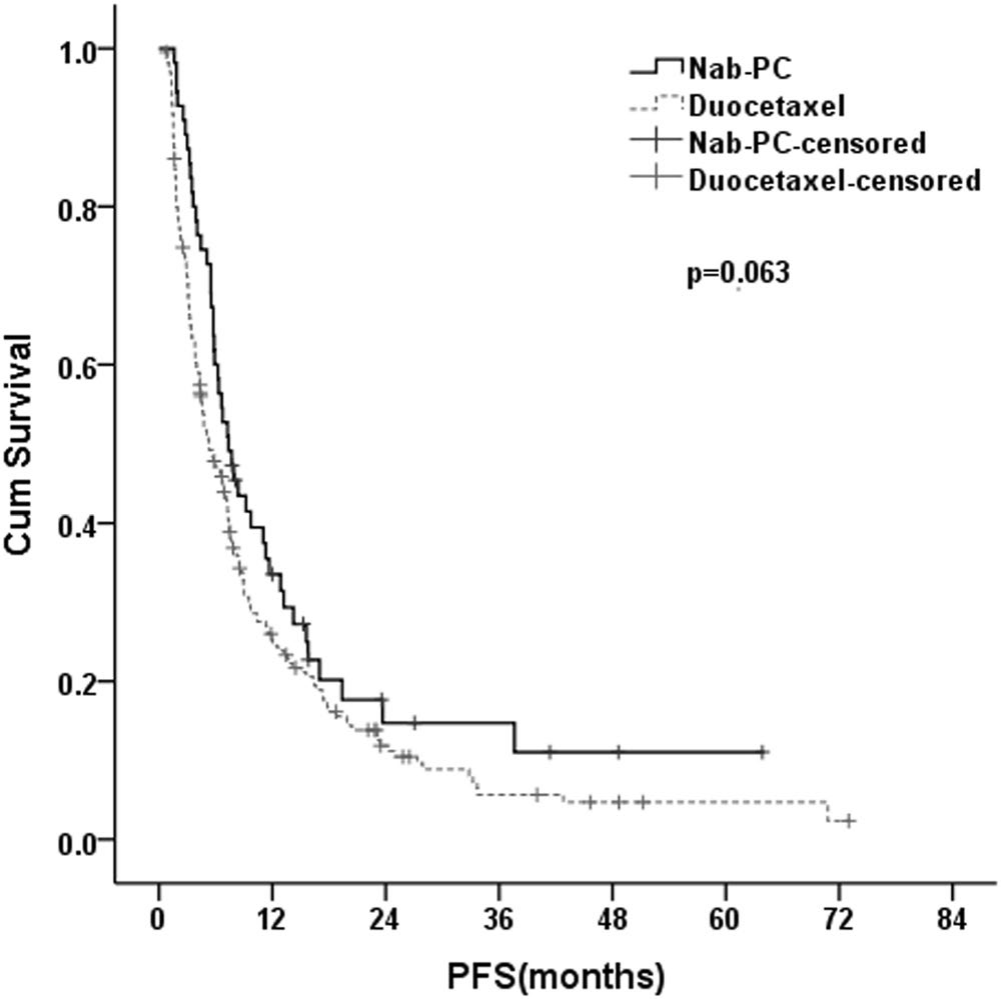
Kaplan–Meier curves showing progression-free survival for the patients in the nab-PC group and docetaxel group.

There was approximately 2 months increase in PFS for nab-PC versus docetaxel, although not significantly different (7.4 months *vs* 5.3 months; p = 0.063), suggesting a trend toward improved PFS for nab-PC in patients with advanced NSCLC(Fig. 1).

### Overall Survival

**Figure 2:**
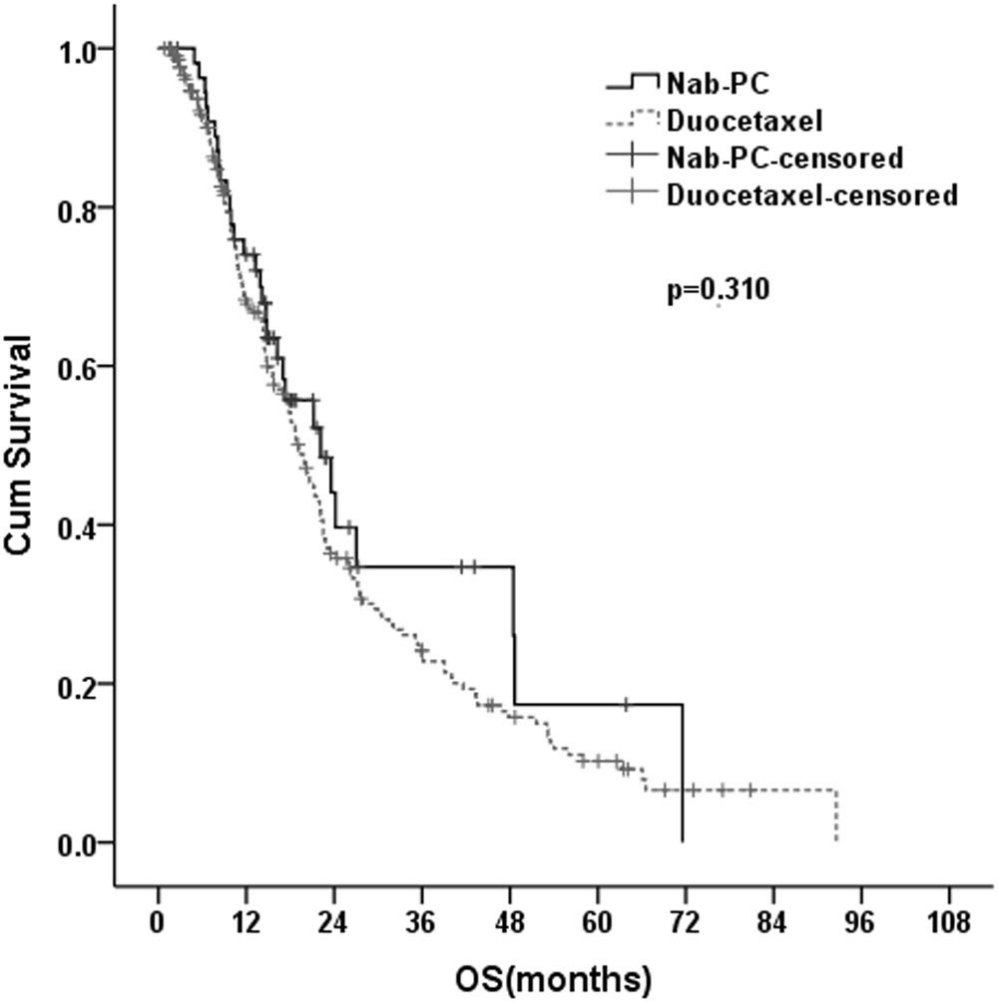
Kaplan–Meier curves showing overall survival for the patients in the nab-PC group and docetaxel group.

There was an about 16% increase in OS for nab-PC versus docetaxel, with median OS of 22.1 months in the nab-PC group and 19.1 months in the docetaxel group in despite of no significant difference (p = 0.31; Fig. 2).

### Safety Results

Generally, toxicities in both groups were well tolerated and manageable. There were significantly less grade ≥ 3 neutropenia in the nab-PC group, but there were less thrombocytopenia and anemia in the docetaxel group (Table 3). The most common hematologic grade ≥ 3 TRAEs with nab-PC and docetaxel were neutropenia (36% *vs* 56%), thrombocytopenia (11% *vs* 3%), and anemia (15% *vs* 2%). The most common non-hematologic grade ≥ 3 TRAEs were fatigue (2% *vs* 3%), nausea/vomiting (7% *vs* 6%), diarrhea (7% *vs* 9%), sensory neuropathy (4% *vs* 5%), myalgia (2% *vs* 1%) and arthalgia (0% *vs* 1%). No treatment-related deaths occurred in each group.

**Table 3:** Most common treatment-related adverse events (Grade ≥ 3).

Adverse Events	Nab-PC (N = 55)	Decetaxel (N = 216)	P^*^ value
	Grade ≥ 3	Grade ≥ 3	
**Hematologic AEs**			
Neutropenia	20(36.4%)	121(56.0%)	0.010
Thrombocytopenia	6(10.9%)	7(3.2%)	0.043
Anemia	8(14.5%)	4(1.9%)	0.000
**Non-hemetologic AEs**			
Fatigue	1(1.8%)	6(2.8%)	1.000
Nausea/Vomiting	4(7.3%)	13(6.0%)	0.975
Diarrhea	4(7.3%)	19(8.8%)	0.927
Sensory neuropathy	2(3.6%)	11(5.1%)	0.922
Myalgia	1(1.8%)	2(0.9%)	0.495
Arthragia	0(0.0%)	2(0.9%)	1.000

Abbreviations: AEs, Adverse Events;

^*^P values were generated by compare the incidence of Grade ≥ 3 AEs between the two groups using Chi-square or Fisher’s exact test.

## Discussion

In this study, we directly compared the efficacy and safety profiles of nab-PC/cisplatin to docetaxel/cisplatin as first-line chemotherapy for patients with advanced NSCLC. The results demonstrated significantly superior efficacy in terms of ORR in the nab-PC/cisplatin group than the docetaxel/cisplatin group. Furthermore, DCR was significantly higher for patients receiving nab-PC (89.1%) than docetaxel (64.4%). The results also indicated a trend toward increased PFS and OS for nab-PC versus docetaxel. These increased efficacy results were accompanied by a reduced toxicity mainly in regard to neutropenia.

This study indicated a significantly more favorable ORR in nab-PC group (47.3%) than docetaxel group (31.9%). Further subgroup analysis revealed that, in patients with squamous histology, nab-PC produced a far significantly higher ORR than docetaxel (58.3% *vs* 29.0%; P = 0.007). However, in patients with non-squamous histology, the response rates were comparable in both groups (38.7% *vs* 34.1%; P = 0.634). In a randomized multicenter study regarding metastatic breast cancer, William *et al*.^37^ also confirmed these results that nab-PC regimen as first-line therapy produced a significantly higher ORR than docetaxel by investigator assessment. In addition, our study indicated that patients with squamous histology responded much better to nab-PC than patients with non-squamous histology (58.3% *vs* 38.7%). These findings were confirmed by a ramdomized multicenter phase III clinical trial^22^ reporting an ORR of 41% in squamous NSCLC and 26% in non-squamous NSCLC treated with nab-PC as first-line therapy. In general, the results of ORR of nab-PC in present study were comparable to previous studies^17, 21–23^.

Similarly, the DCR in nab-PC group is significantly higher than that in docetaxel group, especially in the patients with squamous cell histology. These results echoed the results of a previous study^37^ and strengthen the evidence of better antitumor activity for nab-PC than docetaxel.

To sum up, the PFS of nab-PC and docetaxel in this study was comparable with previous studies^5, 7–14, 17, 22, 23, 38^. Patients in nab-PC group had a trend toward significantly improved PFS (7.4 months), compared with those in docetaxel group (5.3 months), but this didn’t reach statistical significance. In metastatic breast cancer, a study^37^ reported that nab-PC, as first-line therapy, significantly prolonged the PFS by about 5 months compared to docetaxel. A prospective study was needed to confirm whether nab-PC could produce a better PFS for patients with advanced NSCLC than docetaxel in first-line setting.

The OS of nab-PC and docetaxel are comparable, with a 16% improvement for OS favoring the nab-PC group, though not significantly different (22.1 months *vs* 19.1 months; p = 0.31). In metastatic breast cancer, a study^39^ reported that nab-PC, as first-line therapy, significantly prolonged the OS compared to docetaxel. While comparing the data of our study to the randomized multicenter PhaseIII study^22^, we observed that the patients’ survival for nab-PC were better in our study (22.1 months *vs* 12.1 months). Several possible causes following may account for the better OS in this study. First, the participants in this study had more favorable baseline conditions, with fewer patients older than 70 years (10% *vs* 14%), more patients with good performance status (ECOG ≤ 1) (33% *vs* 26%) and less stage IV patients (65% *vs* 79%), compared with the patients in the phase III study, which therefore may contribute to improved survival in our study. Second, most patients in our study received monotherapy or combined therapies after disease progression. About 20% of patients received target therapy directed to driver mutation gene, 4% of patients received immunotherapy as sequential treatment after disease progression. Additionally, most patients received multi-line chemotherapy. Third, most patients received the treatment of Chinese traditional medicine after disease progression.

The toxicity of nab-PC and docetaxel were both acceptable and manageable. The rates of grade ≥ 3 TRAEs were higher in the docetaxel group for neutropenia, compared with nab-PC, but the thrombocytopenia and anemia were higher in nab-PC than docetaxel. Other adverse events were comparable between the nab-PC and docetaxel groups.

The efficacy and safety results of the present study reconfirmed previous results. A previous preclinical study demonstrated nab-PC had a superior antitumor efficacy compared with docetaxel^36^. A phase II study^37^ concerning metastatic breast cancer, demonstrated a significantly prolonged PFS and OS for nab-PC compared to docetaxel in first-line setting. The enhanced efficacy and reduced toxicity with respect to neutropenia may be associated with the more effective intracellular delivery of paclitaxel through the albumin-based nanoparticle technology.

As to limitations, this retrospective study was a single center study involving limited sample size. More well-designed prospective studies to directly compare the efficacy and safety of nab-PC with docetaxel are warranted.

## Conclusion

This study demonstrated that in patients with untreated advanced NSCLC, administration of nab-PC/cisplatin as a first-line therapy resulted in a significantly improved ORR versus docetaxel/cisplatin. Additionally, non-significant improved PFS and OS in favor of the nab-PC group were observed. The nab-PC regimen produced less severe neutropenia compared with docetaxel. This study provided evidences for a new alternative treatment option for patients with previously untreated advanced NSCLC.

## Patients and Methods

### Patients

A study on patients with advanced NSCLC was initiated with valid approval from the Ethics Committee at General Hospital of Chinese PLA in Beijing and all methods were performed in accordance with relevant guidelines and regulations. Patients with advanced NSCLC, who were treated with either nab-PC or docetaxel combined with cisplatin were retrospectively reviewed from 2012 to 2016. Written informed consent was provided by each patient before the treatment.

### Inclusion criteria

The inclusion criteria were: (1) histologically or cytologically confirmed non-resectable stage IIIB (with or without pleural effusion) or stage IV NSCLC; (2) Eastern Cooperative Oncology Group (ECOG) performance status of 0 to 1; (3) age ≥18 years; (4) at least one measurable disease according to Response Evaluation Criteria in Solid Tumor (RECIST); (5) no prior chemotherapy for advanced NSCLC, but prior neo-adjuvant or adjuvant chemotherapy was allowed; (6) no concurrent immunotherapy; (7) no other cancer.

### Chemotherapy regimens

The nab-PC/cisplatin regimen included nab-PC on days 1 and 8 (130 mg/m^2^,iv drop) and cisplatin on day 1 (75 mg/m^2^,iv drop) q3w. The Docetaxel/cisplatin regimen included docetaxel on day 1 (75 mg/m^2^, iv drop) and cisplatin on day 1 (75 mg/m^2^,iv drop) q3w.

### Assessment of Efficacy and Safety Endpoints

The primary efficacy endpoint was ORR in present study. The secondary efficacy endpoints were DCR, PFS and OS. The Treatment efficacy was evaluated according to the Response Evaluation Criteria in Solid Tumors (RECIST) and classified into complete response (CR), partial response (PR), stable disease (SD), and progressive disease (PD). ORR was confirmed complete response (CR) and/or partial response (PR). DCR was calculated by adding up CR, PR, and SD (≥16 weeks). PFS was defined as the interval from the date of treatment initiation to the date of disease progression, or death caused by any reason, or patient censorship at the last follow-up. OS was defined as the interval from the date of treatment initiation to the date of death or patient censorship at the last follow-up. The safety end point was treatment-related adverse events (TRAEs), evaluated by the National Cancer Institute Common Toxicity Criteria version 3.0 and classified as degree 0 (none), degree I (mild), degree II (moderate), degree III (severe), and degree IV (life-threatening).

### Statistical analysis

The patients’ characteristics were described by continuous variables and/or categorical variables. Continuous variables were compared by T test or rank-sum test and categorical variables were compared by Chi-square or Fisher’s exact test. The ORR and DCR were summarized by the number and percentage of patients, odds ratio and 95% CIs of the rates. Treatment group differences in ORR and DCR were tested by Chi-square test. PFS and OS were measured by the Kaplan–Meier method and compared using log-rank tests. Treatment group differences in TRAEs were evaluated by Fisher’s exact test or Chi-square test. P < 0.05 was considered as statistically significant. SPSS 21.0 software was used for statistical analysis.

### Data availability

The datasets generated during and/or analysed during the current study are available from the corresponding author on reasonable request and with permission of the Chinese PLA General Hospital.
